# Multimodal image registration for liver radioembolization planning and patient assessment

**DOI:** 10.1007/s11548-018-1877-5

**Published:** 2018-10-22

**Authors:** Nadine Spahr, Smita Thoduka, Nasreddin Abolmaali, Ron Kikinis, Andrea Schenk

**Affiliations:** 1 Fraunhofer Institute for Medical Image Computing, MEVIS, Lübeck, Germany; 2 Department of Radiology, Städtisches Klinikum Dresden, Dresden, Germany; 3 Medical Image Computing, University of Bremen, Bremen, Germany; 4 Surgical Planning Laboratory, Brigham and Women’s Hospital, Boston, MA USA; 5 Harvard Medical School, Boston, MA USA

**Keywords:** Image registration, Multimodality, Liver, Radioembolization

## Abstract

**Purpose:**

Multimodal imaging plays a key role in patient assessment and treatment planning in liver radioembolization. It will reach its full potential for convenient use in combination with deformable image registration methods. A registration framework is proposed for multimodal liver image registration of multi-phase CT, contrast-enhanced late-phase T1, T2, and DWI MRI sequences.

**Methods:**

A chain of four pair-wise image registrations based on a variational registration framework using normalized gradient fields as distance measure and curvature regularization is introduced. A total of 103 cases of 35 patients was evaluated based on anatomical landmarks and deformation characteristics.

**Results:**

Good anatomical correspondence and physical plausibility of the deformation fields were attained. The global mean landmark errors vary from 3.20 to 5.36 mm, strongly influenced by low resolved images in *z*-direction. Moderate volume changes are indicated by mean minimum and maximum Jacobian determinants of 0.44 up to 1.88. No deformation foldings were detected. The mean average divergence of the deformation fields range from 0.08 to 0.16 and the mean harmonic energies vary from 0.08 to 0.58.

**Conclusion:**

The proposed registration solutions enable the combined use of information from multimodal imaging and provide an excellent basis for patient assessment and primary planning for liver radioembolization.

## Introduction

Selective internal radiation therapy (SIRT) is a type of brachytherapy used in interventional radiology to treat unresectable tumors of the liver, such as hepatocellular carcinoma. The therapy takes advantage of the different supply characteristics of normal liver and liver tumors. The liver parenchyma is perfused mainly by the portal vein and only to a smaller proportion by the hepatic artery. Liver tumors are usually supplied by arterial vessels [6]. Therefore, radioembolization of liver tumors is performed by administering yttrium-90(Y-90)-labeled microspheres to tumor-feeding arterial vessels, targeting the tumor [16]. The microspheres embolize the capillaries and irradiate the surrounding tumor tissue and potentially also surrounding parenchymal regions. Careful treatment planning is required for a successful patient outcome and sparing of normal liver tissue.

**Fig. 1:**
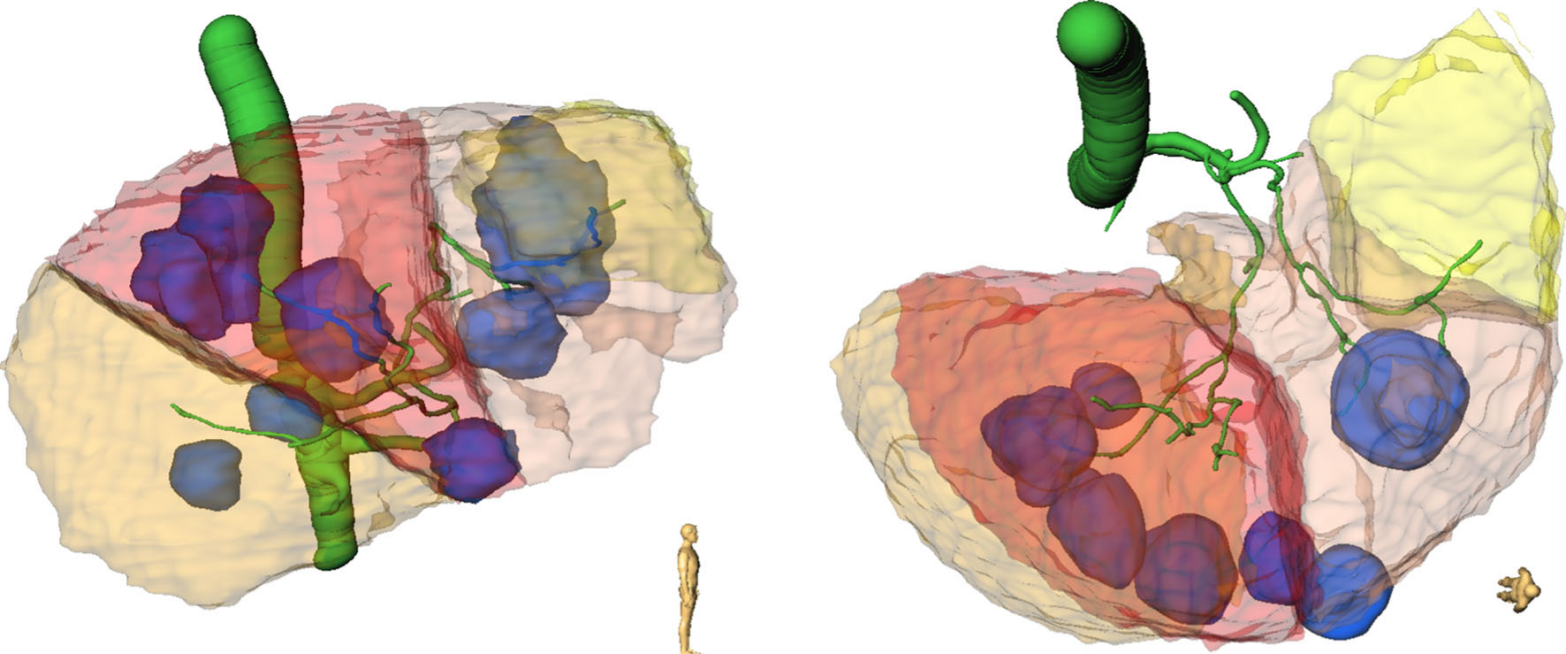
Three-dimensional overview of structures, being relevant for treatment planning, visualized in the same image domain. The liver boundary and lesions were segmented in CE T1W. The T2 was used to distinguish between tumors (blue) and cysts (not shown). Arterial liver vessels (green) were segmented in HA CT. They provide the starting point for the catheter-based radioembolization treatment and for the analysis of arterial supply areas (yellow, ocher, orange, red). A low remaining liver function in non-treated areas is an exclusion criterion. It is assessed via DWI

Several image modalities are involved in the primary planning process of radioembolization . The multimodal images will influence the patient assessment, which is related to whether the patient-individual liver anatomy allows to successfully perform SIRT. Also the treatment plan, which addresses the issue of an effective and efficient treatment, is strongly influenced by them [6]. Multi-phase contrast-enhanced computer tomography (CT) images, in particular the hepatic venous CT phase (PV CT), and/or dynamic magnetic resonance (MR) images, e.g., contrast-enhanced T1-weighted (CE T1W) images, are performed for volume and tumor burden calculation [14]. They allow segmentations of the normal liver parenchyma and a clear delineation of all liver lesions to be present. Additional T2-weighted MR imaging protocols are helpful in lesion characterization, e.g., in order to differentiate between tumors and cysts [35]. The tumors will be targeted by the therapy, whereas cysts are not of particular interest in radioembolization treatment planning. The hepatic arterial CT (HA CT) gives an overview of the patient-individual arterial vessels, which provide potential locations for Y-90 administration. Diffusion-weighted imaging (DWI) adds valuable information on local liver function. All these imaging modalities make a particular contribution to the patient’s individual organ and function, cf. Fig. 1, but it lacks an overall picture. Besides the use of spatial information fusion for patient assessment, SIRT dosimetry, and advanced treatment planning will benefit from this framework to be able to provide a better patient-specific estimation of the treatment biodistribution. As a future perspective, the introduced image modalities provide the basis for methods modeling the particle distribution, which may replace the controversially discussed MAA-SPECT. In order to enable the combined use of information from the different image modalities, image registration techniques are required [13].

Methods for multimodal CT-T1W liver image registration [11, 28, 31], registration of different MR sequences [3, 33], as well as liver CT registration methods [9, 19] can be found in the literature, whereas a dedicated image registration framework tailored to all introduced image modalities in the context of liver radioembolization has not been demonstrated so far. This paper aims at presenting an image registration framework that enables deformable image registration of HA CT, PV CT, CE T1W, T2 and DWI. To the authors’ knowledge, this is the first attempt of an evaluation for multimodal registration in radioembolization including landmark and deformation field analyses.

## Materials and methods

The image registration framework should enable deformable registration of five different image modalities (HA CT, PV CT, CE T1W, T2, DWI) relevant in primary liver radioembolization planning. The goal is to introduce one registration framework that is capable of performing all required registrations by adapting only the parameterization. In order to demonstrate the performance of the methods in terms of spatial accuracy and physical plausibility, the image registration methods are evaluated in detail based on anatomic landmarks and based on deformation field characteristics in a total of 103 cases of image pairs of 35 patients.

### Image data

Routine image data from 35 patients, who underwent radioembolization treatment at Städtisches Klinikum Dresden, Germany, were retrospectively analyzed. This center performs multi-phase CT and contrast-enhanced MRI as standard imaging protocol.

The two-phase contrast-enhanced liver CTs were acquired on a GE LightSpeed VCT (GE Healthcare). The scan parameters were: collimation 0.625 mm, pitch 0.984, rotation time 0.5 s, voltage 80 kV, current 320–680 mA. The MR images were performed on a GE Signa HDxt 1.5T MRI system (GE Healthcare). CE T1W imaging was performed after bolus injection of gadolinium ethoxybenzyl diethylenetriamine pentaacetic acid (Primovist, Bayer Schering Pharma AG) at a rate of 2 ml/s by a high-resolution sequence in breath hold. In the following, we consider the late-venous phase only, which is acquired 15 min after bolus injection. Repetition time, echo time, matrix, field of view, section thickness, and flip angle vary slightly across the patients for CE T1W, T2 and DWI. Table 1 gives an overview of the mean in-plane voxel size, the mean slice thickness, and the detector width of the CT scanner.

**Table 1:** Characteristics of images and evaluation data

Modalities		Data characteristics				
Reference image	Template image	Number of cases	Mean number of landmarks per case	Mean template in-plane voxel size [mm]	Mean template slice thickness [mm]	CT detector width [mm]
HA CT	PV CT	22	9	0.73	0.55	0.625
PV CT	CE T1W	21	8	1.17	2.53	n/a
CE T1W	T2	31	10	0.80	6.19	n/a
T2	DWI	29	9	1.64	6.48	n/a

### Image registration framework

**Fig. 2:**
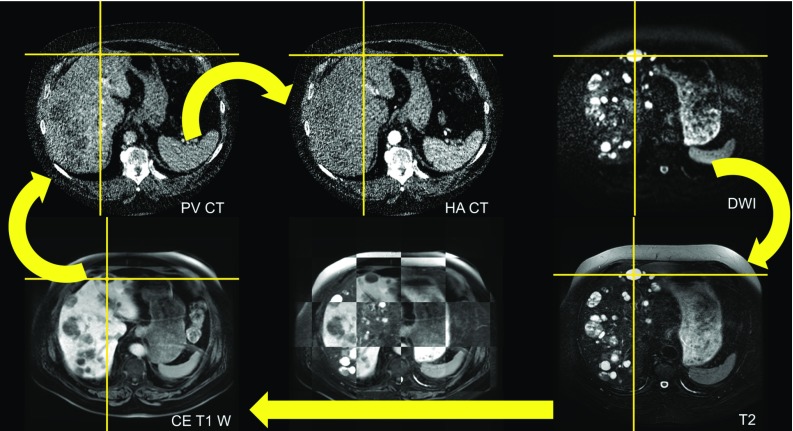
Overview of the image modalities and the proposed registration scheme. Arrows indicate the registration image pairs. The deformable registration results should enable spatial correlation throughout the whole image volumes as indicated by position synchronization of cross-hairs and the checkerboard overlay. This approach focusses on image-based structures, e.g., liver lesions in CE T1W and T2. A direct comparison of HA CT and T2 seems infeasible due to missing image- or landmark-based similarities inside the liver

A preliminary consideration is that the central element of the deformable image registration framework for primary radioembolization planning should be one of the imaging modalities recommended for therapy planning [20], the HA CT or late-venous phase of the CE T1W. On the other hand, we have to overcome the challenge of registering images from multiple modalities, showing different characteristics of the biological tissues. The degree of similarity differs among the modalities of interest, cf. Fig. 2. To aim for the fusion of image-based structures, e.g., liver, tumor or vessel segmentations, an image-based registration framework appears appropriate. Hence, we propose a chain of four image registrations: HA CT-PV CT, PV CT-CE T1W, CE T1W-T2, and T2-DWI. The order of pair-wise registrations was selected in such a way that modalities, emphasizing similar anatomical structures of the liver, are registered directly. This pair-wise approach provides the opportunity to evaluate the accuracy based on landmarks of the anatomical structures visible in both datasets. Figure 2 sketches the proposed registration scheme and emphasizes image-based similarities. In the following, the variational model used for pair-wise registration is introduced, which is based on [17].

#### Variational image registration

Consider $\mathcal {R}:\mathbb {R}^{3}\rightarrow \mathbb {R}$ as the fixed reference image and $\mathcal {T}:\mathbb {R}^{3}\rightarrow \mathbb {R}$ as the moving template image with compact support in domain ${\varOmega }\subset \mathbb {R}^{3}$. The goal of image registration is to find a transformation $y:{\varOmega }\rightarrow \mathbb {R}^{3}$ such that the deformed template image $\mathcal {T}\left( y\right)$ is similar to $\mathcal {R}$. This is modeled by an optimization problem with the objective function $\mathcal {J}$,

1 $$\begin{aligned} \mathcal {J}=\mathcal {D}\left( \mathcal {R},\mathcal {T}\left( y\right) \right) +\alpha \cdot \mathcal {S}\left( y\right) \rightarrow \min . \end{aligned}$$

In this variational model, $\mathcal {D}$ is a distance measure, describing image similarity, and $\mathcal {S}$ is a regularizer, penalizing irregular deformations. $\alpha$ is a regularization parameter, weighting data fit and deformation regularity.

Due to the multimodality of the image registration problem, the normalized gradient fields [10] distance measure is chosen. The underlying assumption is that the images are pair-wise comparable by their image gradients rather than image intensities. Therefore, the distance measure $\mathcal {D}$ is given by2with  and . $\rho$, $\tau \ge 0$ are the so-called edge parameters of the reference and template image, respectively. They specify relevant image gradients and image noise. Deformation regularity is pursued using the curvature regularizer $\mathcal {S}$ [7]

3 $$\begin{aligned} \mathcal {S}\left( y\right) :=\frac{1}{2}\int \limits _{{\varOmega }}\sum \limits _{l=1}^{3}\left\| {\varDelta }u_{l}\right\| ^{2}\text {d}x, \end{aligned}$$

penalizing the Laplacian of the deformation components but ignoring affine linear transformations. In the case of the PV CT-CT T1W image registration, we added a term for volume regularization $\gamma \cdot \mathcal {V}\left( y\right)$ to the objective function [27], where the volume regularization is given by

4 $$\begin{aligned} \mathcal {V}\left( y\right) := \int \limits _{{\varOmega }}\psi \left( \text {det}\nabla y\left( x\right) \right) \text {d}x \end{aligned}$$

with weighting function $\psi \left( t\right) :=\left( t-1\right) ^2/t$ for $t>0$ and $\psi \left( t\right) :=\infty$ else. Empirically chosen registration parameters are summarized in Table 2.

**Table 2:** Overview of registration parameters

Registration method	$\rho$	$\tau$	$\alpha$	$\gamma$
HA CT-PV CT	5	5	10	0
PV CT-CE T1W	1	1	100	$1\times 10^{-3}$
CE T1W-T2	0.5	0.5	100	0
T2-DWI	5	5	50	0

The optimization problem is solved in a discretize-then-optimize scheme [23] using a quasi-Newton L-BFGS optimizer [24].

### Image registration evaluation

Each pair-wise registration is evaluated based on landmark measurements and the analysis of the deformation fields, providing an idea of the overall performance of the image registration algorithms.

#### Landmark-based analysis

In order to evaluate the image registration performance, the correspondence of anatomical landmarks in the images to be registered is one key criterion. Therefore, all images to be evaluated were divided into two groups and two experienced radiological technicians were asked to manually define ten corresponding landmarks in each reference and template image pair of one group. An in-house annotation software was used in order to perform this task [30]. Well-defined positions were selected as landmarks, which are anatomically relevant and visible in both images. We only consider landmarks inside the liver that specify the same anatomical position in both images. Due to poor image contrast or breathing artifacts, the definition of landmarks was challenging and it was not possible to define ten landmarks in all cases. The integer part of the mean number of landmarks for each method is given in Table 1. The related standard deviation is 0 for CE T1W-T2 and 1 in the other cases.

The selected landmarks were used for the evaluation of the image registration methods. As a measure of registration accuracy, we calculated the mean landmark error. It was calculated by the euclidean distance between the manually defined landmark and the transformed landmark position. In addition, we individually analyzed the landmark error for the *x*-, *y*-, and *z*-component. Therefore, the impact of a lower image resolution in *z*-direction compared to the *x*- and *y*-direction was investigated.

#### Deformation field analysis

The deformation at a specified position $x \in \mathbb {R}^3$ is given by $y(x) = x+u\left( x\right)$, with spatial displacement *u*. One criterion to be considered in order to evaluate the physical behavior of the deformation field is the determinant of the Jacobian matrix of the deformation field [21]. It provides information about volumetric changes and transformation consistency. A Jacobian determinant equal to one indicates volume preservation. A Jacobian determinant greater than one specifies volume increase; a value between zero and one specifies volume decrease. A negative value indicates foldings of the deformation field and a physically implausible deformation. Hence, one aims for a positive determinant of the Jacobian. We investigated the number of foldings of the deformation field and the mean minimum and maximum Jacobian determinants.

Volume control or compressibility can be expressed by the divergence of the displacement field [21]. From a physical point of view, it represents the volume density of the outward flux. We investigated the average absolute divergence of the calculated deformation fields.

The smoothness or regularity of the deformation field can be quantified by the harmonic energy $\text {HE}$. It is defined as the average over all voxels *N* of the squared Frobenius norm of the Jacobian of the displacement field on spatial domain ${\varOmega }\subset \mathbb {R}^3$ [26, 32, 34],

5 $$\begin{aligned} \text {HE} = \frac{1}{N} \sum \limits _{{\varOmega }}^{} \left||\nabla u \right||^2_{F}, \end{aligned}$$

and is inversely proportional to the smoothness of the deformation field.

Correlations between local distributions of the Jacobian determinant, the divergence, and harmonic energy maps were also investigated by means of the normalized cross-correlation (NCC) [2].

## Results

**Fig. 3:**
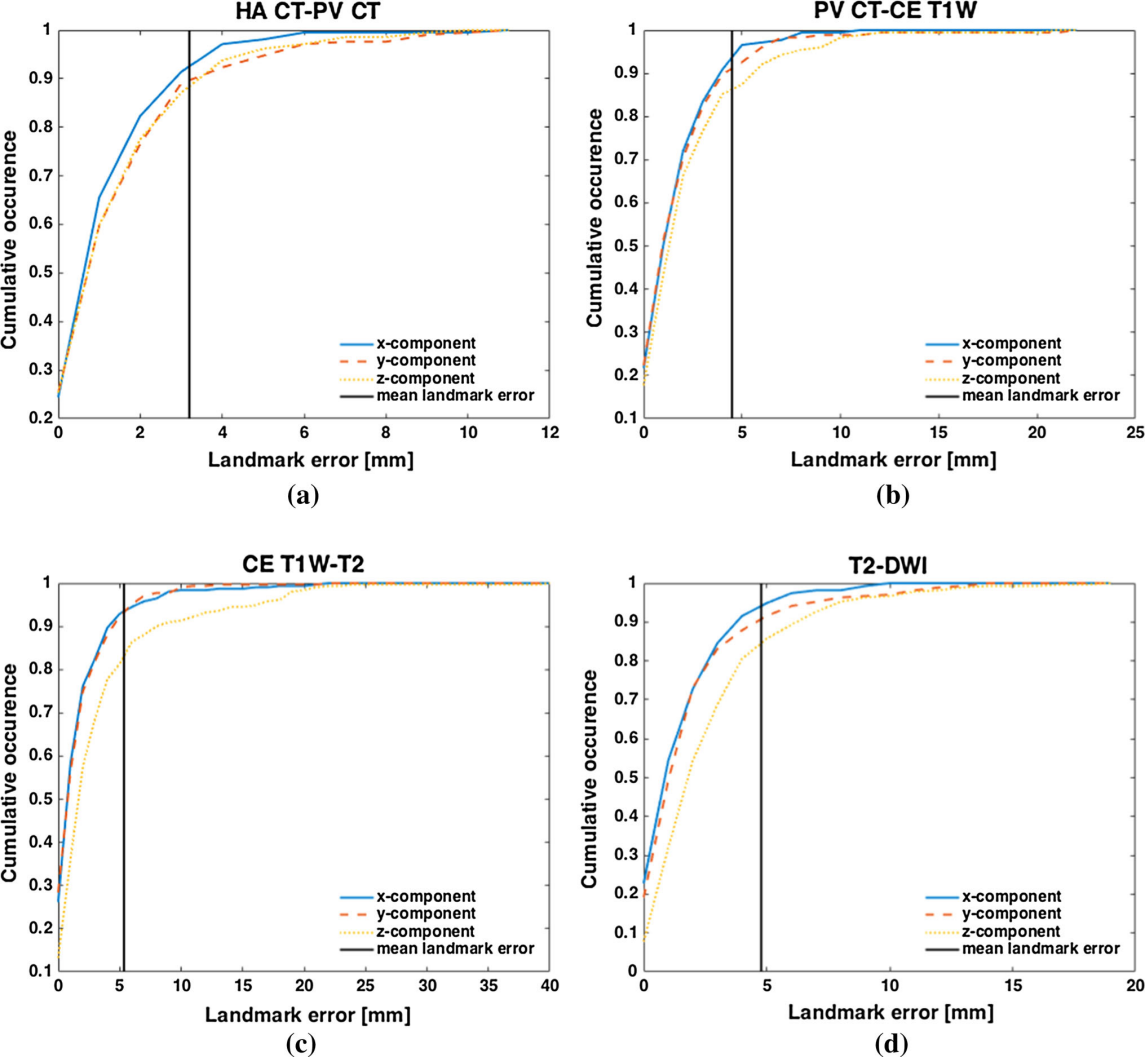
Analysis of the *x*-, *y*-, and *z*-component of the landmark errors for HA CT-PV CT (**a**), PV CT-CE T1W (**b**), CE T1W-T2 (**c**), and T2-DWI (**d**) image registration. Each diagram summarizes the absolute value of component-wise landmark errors from all landmark pairs in all modality-related cases. In addition, the mean landmark error is given by the vertical bar

The results of the landmark-based evaluation of the image registration solutions are summarized in Table 3. The global mean landmark errors are 3.20 mm, 4.49 mm, 5.36 mm, and 4.78 mm for HA CT-PV CT, PV CT-CE T1W, CE T1W-T2, and T2-DWI image registration, respectively. An individual investigation of the *x*-, *y*-, and *z*-component of the landmark errors showed that the *z*-component has the greatest impact on the global mean landmark error, except for the HA CT-PV CT registration solution. The mean landmark errors of the *x*- and *y*-components are quite similar. The diagrams in Fig. 3 visualize the size distribution of landmark errors on a component-wise basis. It underlines the main impact of the *z*-component on the mean landmark error and a similar distribution of errors for the *x*- and *y*-components. The cumulative occurrence indicates that the major amount (> 80%) of landmark errors is smaller than the mean error. Considering the mean template slice thickness, the mean landmark errors of CE T1W-T2 and T2-DWI image registration solutions are within the mean slice thickness, whereas the mean landmark error in case of HA CT-PV CT and PV CT-CT T1W image registration is clearly larger than the mean template slice thickness.

**Table 3:** Results of the landmark-based evaluation criteria

Modalities		Image property	Mean landmark error [mm]			
Reference	Template	Mean template slice thickness [mm]	Global	*x*-Component	*y*-Component	*z*-Component
HA	PV	0.55	3.20	1.48	**1.74**	1.68
PV	CE T1W	2.53	4.49	1.96	2.11	**2.52**
CE T1W	T2	6.19	5.36	2.05	2.00	**3.76**
T2	DWI	6.48	4.78	1.87	2.20	**3.11**

The spatial component with the highest contribution to the overall landmark error is displayed in bold

**Fig. 4:**
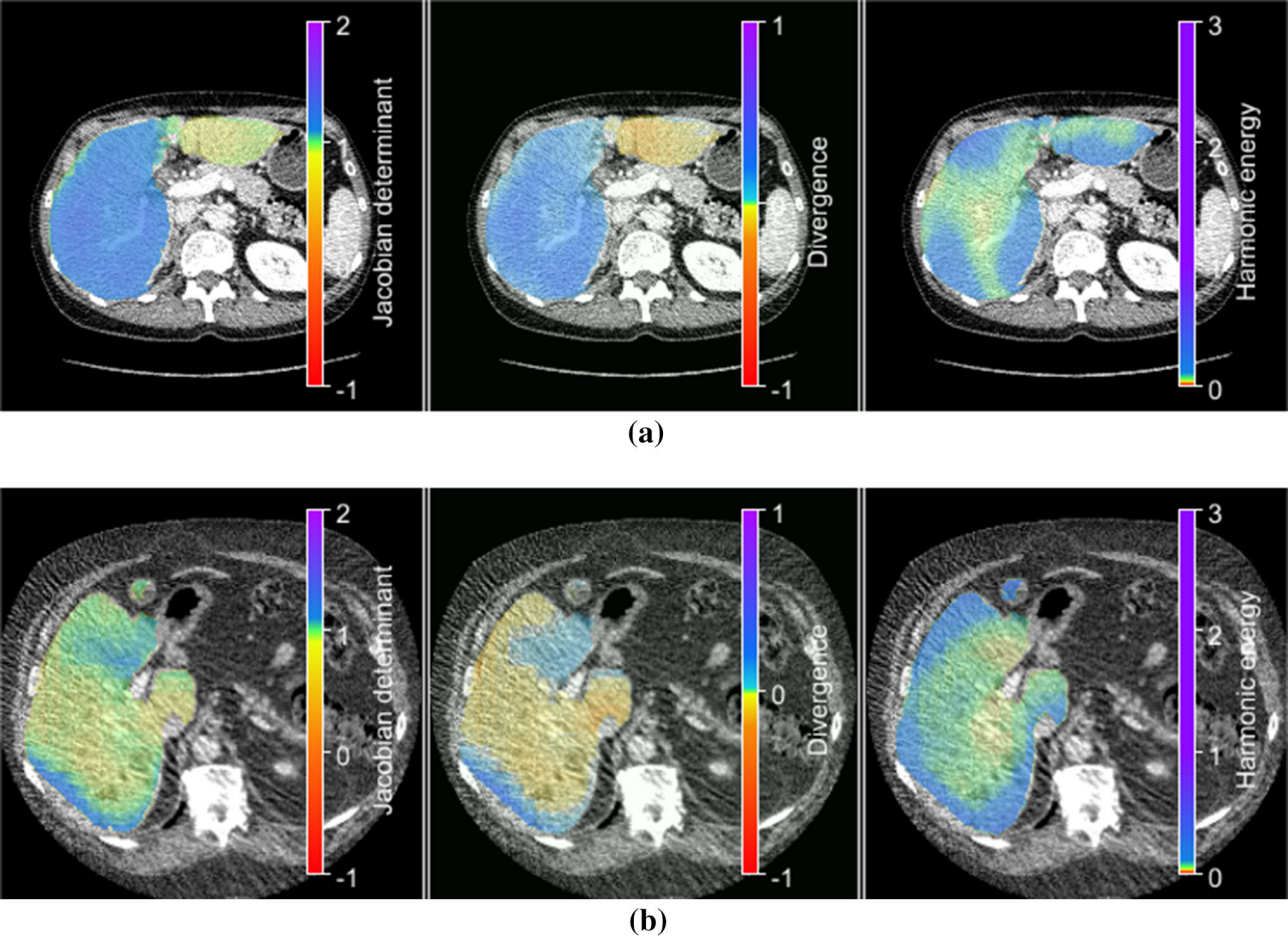
Visualization of the local distributions of the Jacobian determinant (first column), the divergence (second column) and harmonic energy maps (last column) inside the liver as color-overlay on the PV CT images of two patients (**a**, **b**). The displayed maps were calculated from the PV CT-CE T1W deformation fields. Range of Jacobian determinant, mean absolute divergence, and harmonic energy are given by the color, respectively. **a**$J\in (0.54,1.57),\hbox { mean}(|\hbox {div(y)}|)=0.09,\hbox { HE}=0.09$. **b**$J\in (0.49,1.91),\hbox { mean}(|\hbox {div(y)}|)=0.15,\hbox { HE}=0.14$

**Fig. 5:**
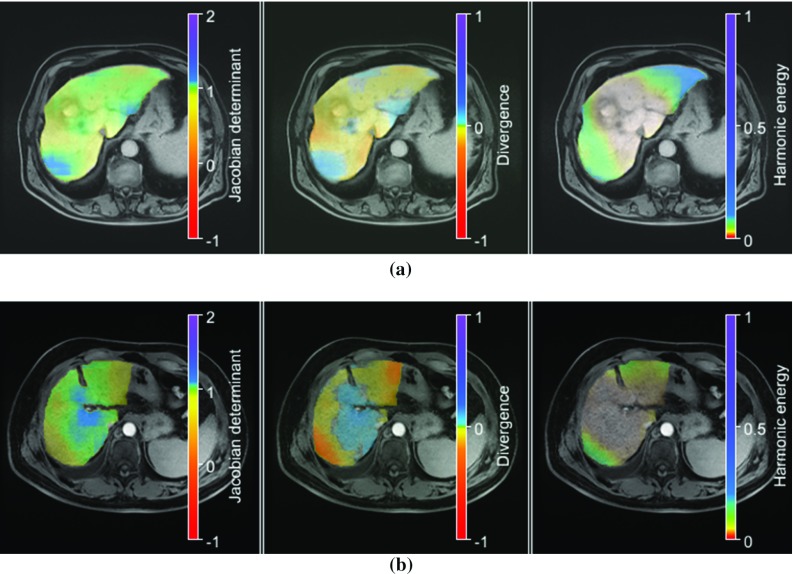
Visualization of the local distributions of the Jacobian determinant (first column), the divergence (second column) and harmonic energy maps (last column) inside the liver as color-overlay on the CE T1W images of two patients (**a**, **b**). The displayed maps were calculated from the CE T1W-T2 deformation fields. Range of Jacobian determinant, mean absolute divergence, and harmonic energy are given by the color bar, respectively. **a**$J\in (0.27,1.55),\hbox { mean}(|\hbox {div(y)}|)=0.24,\hbox { HE}=0.14$. **b**$J\in (0.45,1.39),\hbox { mean}(|\hbox {div(y)}|)=0.20,\hbox { HE}=0.09$

The mean values of the minimum and maximum Jacobian determinants indicate moderate volume changes with maximum volume changes in approximately halving or doubling of cell volume, see Table 4. The deformation fields do not have any foldings. The mean average divergence is rather low and the mean harmonic energy is 0.08, 0.25, 0.12, and 0.58 for HA CT-PV CT, PV CT-CE T1W, CE T1W-T2, and T2-DWI image registration, respectively. We further investigated the local distribution of the Jacobian determinant, the divergence, and the local harmonic energy maps of the deformation fields by mean normalized cross-correlation coefficients calculated from all registration results. A good spatial correlation was demonstrated in case of the Jacobian determinant and the divergence, except for HA CT-PV CT, cf. Table 5. A correlation of the local distribution of the Jacobian determinant and the harmonic energy maps or between divergence and local harmonic energy maps was not found. In case of the PV CT-CE T1W image registration and the CE T1W-T2 image registration, we also visually analyzed the deformation inside the segmented liver and found that a majority of cases show a low harmonic energy in the middle of the liver and larger harmonic energies at the liver periphery, cf. Figs. 4 and 5.

**Table 4:** Results of deformation field analysis

Modalities		Deformation field measures				
Reference image	Template image	Mean min. *J*	Mean max. *J*	Number foldings	Mean HE	Mean average $|\text {div}(u)|$
HA CT	PV CT	$0.44\pm 0.17$	$1.79\pm 0.22$	0	$0.08\pm 0.07$	$0.08\pm 0.03$
PV CT	CE T1W	$0.50\pm 0.09$	$1.74\pm 0.39$	0	$0.25\pm 0.28$	$0.16\pm 0.08$
CE T1W	T2	$0.43\pm 0.18$	$1.65\pm 0.33$	0	$0.12\pm 0.15$	$0.14\pm 0.07$
T2	DWI	$0.48\pm 0.11$	$1.88\pm 0.30$	0	$0.58\pm 0.27$	$0.11\pm 0.03$

Mean values and the standard deviation are given

**Table 5:** Correlation between local distributions of the Jacobian determinant, the divergence, and harmonic energy maps

Modalities		Normalized cross-correlation		
Reference	Template	$\hbox {NCC}\left( J, \text {div}\left( u\right) \right)$	$\hbox {NCC}\left( J, \text {HE}\right)$	$\hbox {NCC}\left( \text {div}\left( u\right) , \text {HE}\right)$
HA	PV	$0.62\pm 0.40$	$<0.01\pm 0.02$	$0.37\pm 0.40$
PV	CE T1W	$0.90\pm 0.10$	$0.04\pm 0.04$	$0.05\pm 0.07$
CE T1W	T2	$0.79\pm 0.17$	$0.05\pm 0.13$	$0.23\pm 0.19$
T2	DWI	$0.70\pm 0.30$	$0.02\pm 0.03$	$0.05\pm 0.11$

Mean values and the standard deviation are given

## Discussion

The landmark- and deformation field-based evaluation of the registration methods demonstrate anatomical correspondence and physical plausibility. The results show that the mean landmark errors of the CE T1W-T2 and T2-DWI image registration solutions are within the mean slice thickness and therefore indicate high spatial correspondence. Nevertheless, the definition of three-dimensional landmarks is a very subjective and challenging task causing non-negligible inter-observer errors, which were about 3 mm in the multi-phase CE T1W images [30]. Hence, the mean landmark error in case of the HA CT-PV CT and PV CT-CE T1W image registration might be strongly biased by this phenomenon. The results are comparable to or better than those reported by other groups: In the context of CT-guided liver ablation, a landmark error of $5.3~\pm ~2.5~\hbox {mm}$ was reported for deformable registration of liver CT images with an intra-observer variation of approximately 2 mm [19]. The mean fiducial errors of follow-up CT registration in two commercial products were stated to be 9.3 mm and 11 mm [9], and the investigation of different algorithms for liver CT-MRI registration reported average errors of 3.9, 4.5, and 6.5 mm [1]. For benchmarking purposes, we also evaluated the liver HA CT-PV CT registration on the DIR-Lab reference dataset [4, 5]. We obtained an average landmark error of 1.93 mm, staying below the values of 2.14 mm and 2.07 mm reported by Heinrich et al. [11] and de Senneville et al. [28]. However, the comparison seems unfair, as our method was not specifically adapted to lung image registration. A performance evaluation on the DIR-Lab dataset with adjusted parameters to the demands of lung image registration (e.g., $\alpha =1$, $\rho =\tau =10$) was presented in [18] and an average landmark error of 0.93 mm was reported there.

By an individual investigation of the *x*-, *y*-, and *z*-component of the landmark errors, we confirmed that the *z*-component makes the biggest contribution to the mean error in most cases. This leads to the assumption that the definition of landmarks in low resolved spatial directions is challenging. An investigation of the landmark definition in images with non-isotropic image resolution as well as the analysis of inter- and intra-observer errors might provide further insight into this topic. In addition, the accuracy and precision of the registration would benefit from an optimization of the acquisition geometry, allowing for smaller slice thicknesses. Nevertheless, the analysis was performed on data from clinical routine and experienced radiological technicians performed the definition of landmarks. As the achieved registration accuracy is below the slice thickness, below or close to the inter-observer error, the developed methods seem accurate enough for the intended application.

The analysis of the deformation fields demonstrates the absence of any foldings. Therefore, the deformation fields can be considered plausible in the sense of spatially allowed transformations. For the PV CT-CT T1W registration, this behavior was forced by design, adding the volume constraint. There might be a relation to the specific imaging protocols, as the two-phase CT was acquired in one imaging session and the MRI sequences were also acquired in one session, but on different days. This means that also the type of the expected deformation of the PV CT-CE T1W registration might differ from the other registrations.

The Jacobian determinant and the deformation divergence show moderate volume changes, which was expected in this specific intra-patient liver registration scenario. The range of mean minimum and maximum Jacobian determinants of around 0.45 and 1.7 was determined on the voxel dimension and is consistent with findings on the local nonlinear part of the liver deformation being smaller than 5 mm [12]. The correlation between local distributions of the Jacobian determinant and the divergence indicates that the underlying deformation is rather smooth, or more precisely, that the deformation is differentiable and does not fluctuate significantly at any point [22]. Technical details on this are given in Appendix 6. The low correlation in case of HA CT-PV CT and T2-DWI is caused by some outliers exhibiting no correlation as indicated by the high standard deviation. The smoothness of the deformation is also represented by low harmonic energies. Freiman et al. [8] reported median harmonic energies of approximately 0.13 for local-affine diffeomorphic demons, 0.16 for diffeomorphic demons, and 0.2 for demons in controlled experiments on an abdominal CT atlas and artificially generated ground truth deformations. In comparison, our proposed registration solutions reached similar values even on routine image data, acquired for liver radioembolization. Only in case of the T2-DWI registration, the measured harmonic energy is higher. Lower harmonic energy values in the middle of the liver volume and larger values at the liver periphery indicate higher strains at the liver periphery. This should be further analyzed in subsequent studies as well as the relation to local tissue properties. Paulsson et al. [25] investigated respiratory-induced liver deformation. They also observed greater deformation at the periphery than at the center of the liver. To verify these observations and our conclusion, patient-individual measurements of physical parameters would be required for a reliable comparison [15]. In addition, the relation and impact of sliding liver motion could be considered further. The curvature regularization prohibits non-smooth deformations in the current approach and introduces a bias to the displacement vectors at the liver boundary. Considering sliding liver motion and therefore larger displacement vectors will result in higher harmonic energy values at the liver periphery. In order to investigate the quality and stability of the registration parameters, a systematic evaluation and potential optimization should be developed in a next step. Also the selection and stability of the chosen parameters might need further investigation.

The proposed image registration framework focusses on the fusion of multimodal images, namely HA CT, PV CT, CE T1W, T2, and DWI. Therefore, it helps to provide valuable joint information in order to assess the applicability of liver radioembolization. Regarding the registration evaluation criteria, the intended goal of information fusion has been reached with satisfactory precision. Detailed treatment planning requires the integration of additional modalities like SPECT/CT [16] or a multi-slice multi-gradient echo MR-sequence [29] to estimate the intra-hepatic distribution of SIRT particles for dose planning. Therefore, future work includes the integration of these image modalities into the registration framework. In combination with segmentation algorithms and models for activity and dose calculation, it can provide a whole platform for radioembolization planning.

## Conclusion

A multimodal image registration framework was presented for patient assessment and primary treatment planning for radioembolization of the liver. In order to register the multimodal CT and MR images, a chain of four pair-wise image registrations was introduced based on a variational registration framework using normalized gradient fields as distance measure and curvature regularization. It was evaluated on a total of 103 cases of 35 patients, yielding good anatomical correspondence and physically plausible deformations. This framework provides a basis for patient assessment and primary treatment planning for liver radioembolization.

## Appendix

### Relation between Jacobian determinant and divergence

The Jacobian determinant and the divergence of a deformation field are related to each other which will be emphasized in the following [22]. Therefore, we consider the Jacobian determinant for the deformation *y* and the incremental displacement *u* in a two-dimensional case, which is given by

6 $$\begin{aligned} J = \det \left( \nabla y \right) = \begin{vmatrix} \begin{pmatrix} \frac{\partial y_{1}}{\partial x_{1}} &{} \frac{\partial y_{1}}{\partial x_{2}} \\ \frac{\partial y_{2}}{\partial x_{1}} &{} \frac{\partial y_{2}}{\partial x_{2}} \\ \end{pmatrix} \end{vmatrix} = \begin{vmatrix} \begin{pmatrix} \frac{\partial u_{1}}{\partial x_{1}}+1 &{} \frac{\partial u_{1}}{\partial x_{2}} \\ \frac{\partial u_{2}}{\partial x_{1}} &{} \frac{\partial u_{2}}{\partial x_{2}}+1 \\ \end{pmatrix} \end{vmatrix}. \end{aligned}$$

A closer look at the Jacobian determinant reveals the relation to the divergence:

7 $$\begin{aligned} J = 1 + \underbrace{\frac{\partial u_{1}}{\partial x_{1}} + \frac{\partial u_{2}}{\partial x_{2}}}_{\text {div}\left( u\right) } + \underbrace{\frac{\partial u_{1}}{\partial x_{1}} \frac{\partial u_{2}}{\partial x_{2}} - \frac{\partial u_{1}}{\partial x_{2}} \frac{\partial u_{2}}{\partial x_{1}}}_{\text {high-order term}} \approx 1 + \text {div}\left( u\right) \nonumber \\ \end{aligned}$$

Thus, the divergence is an approximation of the Jacobian determinant for small partial derivatives.
